# Evaluating climbing interventions for Parkinson's disease: a systematic review

**DOI:** 10.3389/fspor.2026.1695888

**Published:** 2026-03-13

**Authors:** Ryan Alexander Smith, Allison Anne Hellman

**Affiliations:** 1 Alternative Medicine and Music for Parkinson's Disease Lab, Department of Kinesiology and Health, Iowa State University, Ames, IA, United States; 2 Biotechnology Program, Department of Biology, Syracuse University, Syracuse, NY, United States

**Keywords:** exercise, gait, movement disorder, neurodegenerative diseases, Parkinson's disese, rehabilitation, sport climbing, wearable sensors

## Abstract

**Introduction:**

Exercise ameliorates symptoms and may modify disease progression in people with Parkinson's disease (PD). Climbing is an increasingly popular form of exercise with characteristics that may be well suited to addressing the symptoms of PD. This systematic review aims to synthesize and evaluate climbing-based intervention studies for PD.

**Methods:**

This review was not registered. A literature search was conducted on July 1, 2025, using Web of Science, PsycINFO, PubMed, and Google Scholar.

**Results and Discussion:**

Five articles representing three studies and 102 distinct participants with PD met these criteria. The risk of bias was evaluated using the 20-point Methodological Quality of Exercise Training Studies Scale. The results of the included articles were combined into a narrative synthesis. Methodologies of the reviewed articles included randomized controlled trials, pilot studies, and feasibility studies. The results suggested that climbing is an acceptable and feasible form of exercise for people with PD. Climbing may also ameliorate Parkinsonian motor symptoms, including symptoms measured using wearable technologies. Participant experiences of climbing and the effects on non-motor symptoms remain under-examined. Major limitations of the current literature are the small number of publications and small respective sample sizes. Future research examining the feasibility and physiological responses to different types of climbing, as well as comparing climbing to other exercise types and treatment approaches, may help clinicians establish recommendations related to therapeutic climbing for people with PD.

## Introduction

Parkinson's disease (PD) is the second most prevalent neurodegenerative disorder worldwide and there will be an estimated nine million people living with PD globally by 2030 (1–4). The motor symptoms of PD include bradykinesia, tremor, rigidity, and postural instability. Non-motor symptoms are also prominent and include cognitive, sensory, affective, and autonomic changes (5, 6).

There is no known cure for PD, and the primary goal of treatment is to manage symptoms (7). Dopamine replacement medication and deep brain stimulation (DBS) are the most common treatments for PD, but do not fully ameliorate all symptoms (8). Pharmacological and surgical interventions remain expensive, cause cognitive and motor side effects, and do not fully impact the progression of the disease (3). Integrative medicine, especially exercise and interdisciplinary therapies, target symptoms and disease progression that may not be effectively addressed by conventional medicine (9, 10).

Exercise is one of few treatments for which there is evidence of disease-modifying effects in people with PD (11, 12). A comprehensive exercise prescription for PD includes aerobic exercise, resistance training, flexibility exercise, and neuromotor exercise (13). People with PD may engage in multiple exercise types including treadmill walking, balance training, weight training, aquatic exercise, boxing, cycling, Nordic walking, dance, stretching, exergaming, and mind-body practices (10, 14–18). Movement-based and exercise therapies often emphasize large movements to counteract bradykinesia and may involve external cueing and modifications to the environment (19).

**Figure 1:**
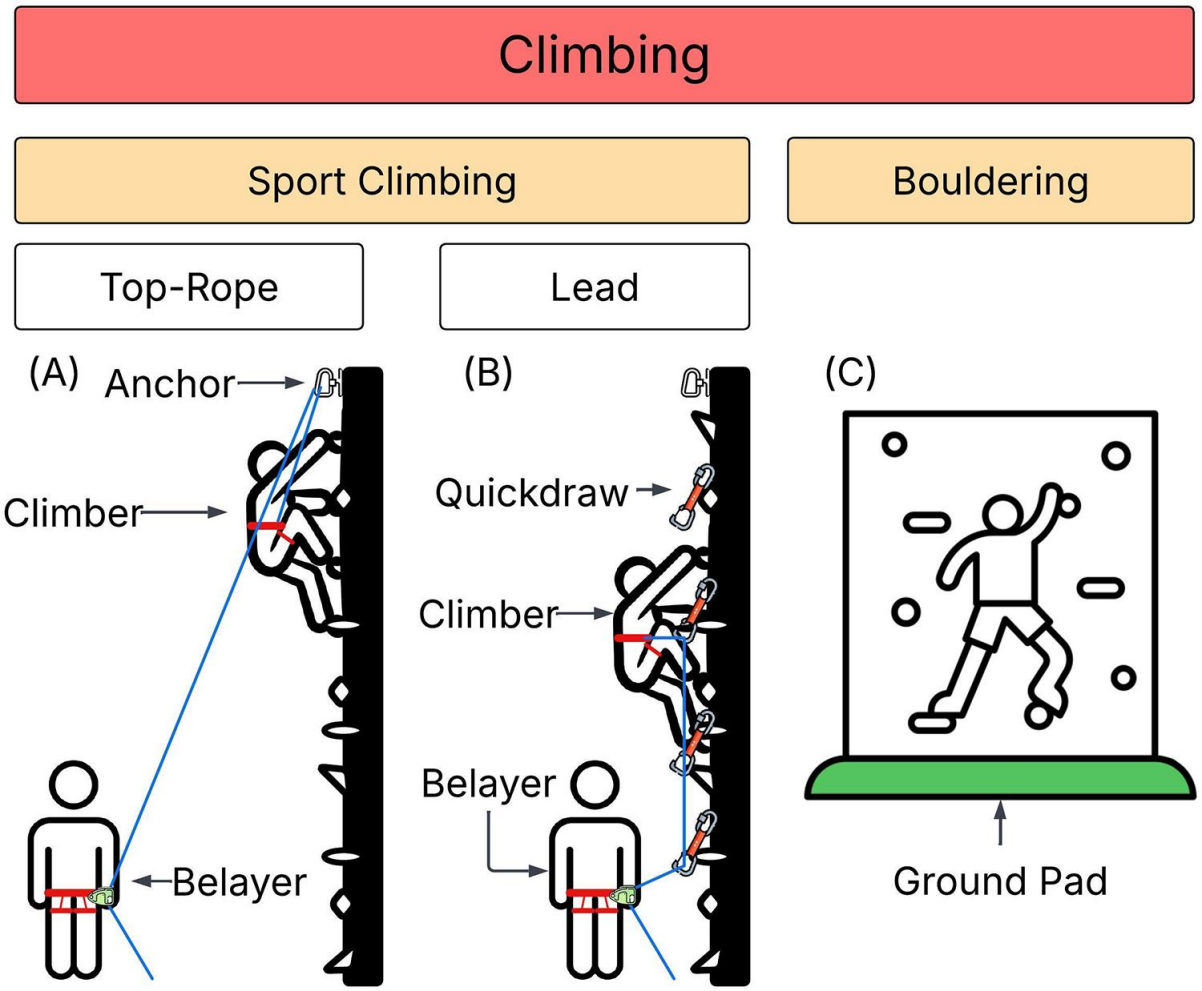
Types of indoor climbing. **(A)** Top-rope is a type of sport climbing in which a rope is attached to the climber and belayer and secured to an anchor at the top of the wall. The belayer takes up slack as the climber progresses, minimizing distance in the case of a fall. **(B)** Lead climbing is a type of sport climbing typically performed by experienced climbers. The climber carries the rope up with them and periodically clips it into quickdraws, which are pieces of protection affixed periodically to the wall. In the event of a fall, the climber falls at least two times the distance between the last quickdraw they clipped and the point at which the rope is tied to their harness. **(C)** Bouldering is performed on low walls without a belayer, harness, or rope system. A protective ground pad is used to slow deceleration and decrease the risk of injury in the case of a fall. Figure 1 was created by the authors using several free icons under the FlatIcons License (56–61).

Climbing is an increasingly popular and accessible form of exercise in which people ascend natural or artificial faces. The term climbing includes a range of sub-types of both outdoor rock climbing and indoor gym climbing. Climbing can also be divided by wall height and safety protection level into sport climbing (SC) and bouldering (20). The sub-disciplines of indoor climbing are presented in Figure 1. These different forms of climbing differently mitigate risks and, as such, researchers have implemented them differently in exercise interventions (21). In SC, a system of rope and harnesses is used to catch falls. Top-rope is the safest sub-type of SC because the rope secured at the top of the climb quickly becomes taut to arrest falls and, as such, is the sub-type of SC most widely used in climbing-based interventions (22). Lead climbing, the other form of SC referenced in Figure 1, is not typically used in therapeutic interventions due to the increased fall distance and expertise needed to lead climb safely. Researchers have examined top-rope interventions to address symptoms of chronic lower back pain, multiple sclerosis, major depressive disorder, attention deficit hyperactivity disorder, cerebral palsy, and cerebellar ataxia (21). In contrast to SC, bouldering involves climbing on low faces using ground pads and sometimes human spotters to prevent injuries in the case of falls (22). Therapeutic applications of bouldering have focused on chronic lower back pain and mental health problems including major depressive disorder (21). Because of the increased fall risk associated with bouldering, top-rope is considered the safest form of indoor climbing (23).

Programs offering climbing interventions for people with PD have received notable media attention—perhaps because of the perception that climbing is an extreme sport that may be risky for people with PD. Despite perceived risks, climbing may be beneficial for people with PD because it engages the four domains recommended in exercise prescriptions—aerobic exercise, strength training, flexibility training, and neuromotor exercise (22). Climbing increases heart rate while simultaneously requiring repeated concentric and isometric contractions, sometimes until failure (20). Additionally, climbing requires various large range-of-motion movements on limited sets of holds, thus necessitating multi-step motor planning (10). Climbing may therefore engage the four exercise types that are recommended for people with PD. Furthermore, climbing may be novel, social, emotionally engaging, and motivating—all of which are factors associated with exercise adherence among older adults (24).

The characteristics of climbing as exercise align with the exercise guidelines for people with PD, and clinicians and researchers are beginning to evaluate the therapeutic effects of climbing in this population. For clinicians to make informed recommendations about climbing as part of an exercise prescription, it is important to understand the current state of the research. To date, there are no published reviews of climbing interventions for PD, nor assessments of the quality of extant publications. Therefore, the purpose of this systematic review is to synthesize and evaluate the literature examining climbing-based interventions for PD, and to identify directions for future inquiry in the field.

## Materials and methods

### Search strategy & inclusion criteria

This review was conducted in accordance with PRISMA guidelines, with slight modifications made to perform narrative synthesis without meta-analysis. This systematic review was not registered, and no protocol paper was published. The search strategy for this review was based on the database combinations for systematic reviews outlined by Bramer et al. (25). Before formal searches were performed, an initial pilot search was conducted in Google Scholar to assess the quantity of publications related to both Parkinson's disease and rock climbing. The search phrase for the pilot search was “Parkinson* AND climb*”. This search primarily returned literature related to stair climbing and research in animal models, which were outside the intended scope of the review. The pilot search also returned a small quantity (<10) of potentially relevant results. Based on the pilot search, the search terms were refined to “Parkinson* AND sport climb*” to capture primarily relevant literature. Database searches were conducted using Web of Science (RRID:SCR_022706), PsycINFO (RRID:SCR_014799), PubMed (RRID:SCR_004846) and Google Scholar (RRID:SCR_008878, first 200 results). The keyword combination “Parkinson* AND sport climb*” was used to search articles published before the search was conducted on July 1, 2025. Two authors collaboratively screened the reports returned using a citation manager. Reference chaining was used to identify additional sources in the reference sections of the included literature.

Abstracts of records were screened for inclusion criteria, and full reports of potentially eligible publications were accessed. Full texts were then screened to remove duplicates and publications that did not meet the full inclusion criteria. Inclusion criteria required that articles be published in a peer-reviewed journal, have a full text available in English or Spanish, present data collected from human participants, include participants with PD, and include quantitative comparison of PD-related measures before and after a climbing-based intervention. There were no additional exclusion criteria, such as disease progression, participant age, and study location. All human subjects study designs were eligible for inclusion; the synthesis and evaluation tools were selected to be flexible to multiple methodologies. All records meeting the inclusion criteria were included in the synthesis.

### Data collection process

Two authors independently read each of the eligible publications and took comprehensive notes on study design, methods, intervention characteristics, and all reported outcomes. Outcomes were characterized by domain, including motor symptoms, balance, locomotion, reaction time, strength, non-motor symptoms, feasibility, and acceptability. The authors then compared the extracted quantitative results and categorizations to resolve discrepancies. No automation was used in the data collection process. Data collection forms are available from the corresponding author upon request.

### Quality assessment

Two authors independently evaluated the methodological and reporting quality of each included article. The quality assessment was conducted using the Methodological Quality of Exercise Training Studies Scale, a rating scale that is flexible to multiple methods and exercise types and has been previously used in systematic reviews of physical activity interventions (26, 27). This scale evaluates constructs that are also used to assess the risk of bias in non-exercise interventions (28). To determine a quality score, each of 10 criteria were scored on a two-point scale—0 = clearly no, 1 = maybe or reported with low quality, 2 = clearly yes. The criteria were: 1) participant inclusion criteria were clearly stated, 2) a control group was included, 3) participants were assigned to groups randomly and groups had equal baselines, 4) the intervention was thoroughly described, 5) dependent variables were defined, 6) assessment protocols were defined and practical, 7) the training duration was practical, 8) statistical tests were applied appropriately, 9) results were presented in detail and include values from statistical tests, 10) conclusions were insightful. Following independent evaluation, the authors met to discuss discrepancies and reach a consensus score. The quality assessment resulted in a total score out of 20 for each article. Total quality and sub-domain scores were presented in a table.

### Synthesis methods

Total quality and sub-domain scores were presented in a table. Results from the collected data were presented in a narrative synthesis organized by outcome domain. Multiple databases were searched, and reference chaining used to minimize the probability of excluding relevant literature. No systematic certainty assessment was conducted due to the limited quantity of extant literature; however, indicators of certainty were included in the quality assessment. Formal assessment of publication bias was not practical due to the small number of publications that met inclusion criteria.

## Results

**Figure 2:**
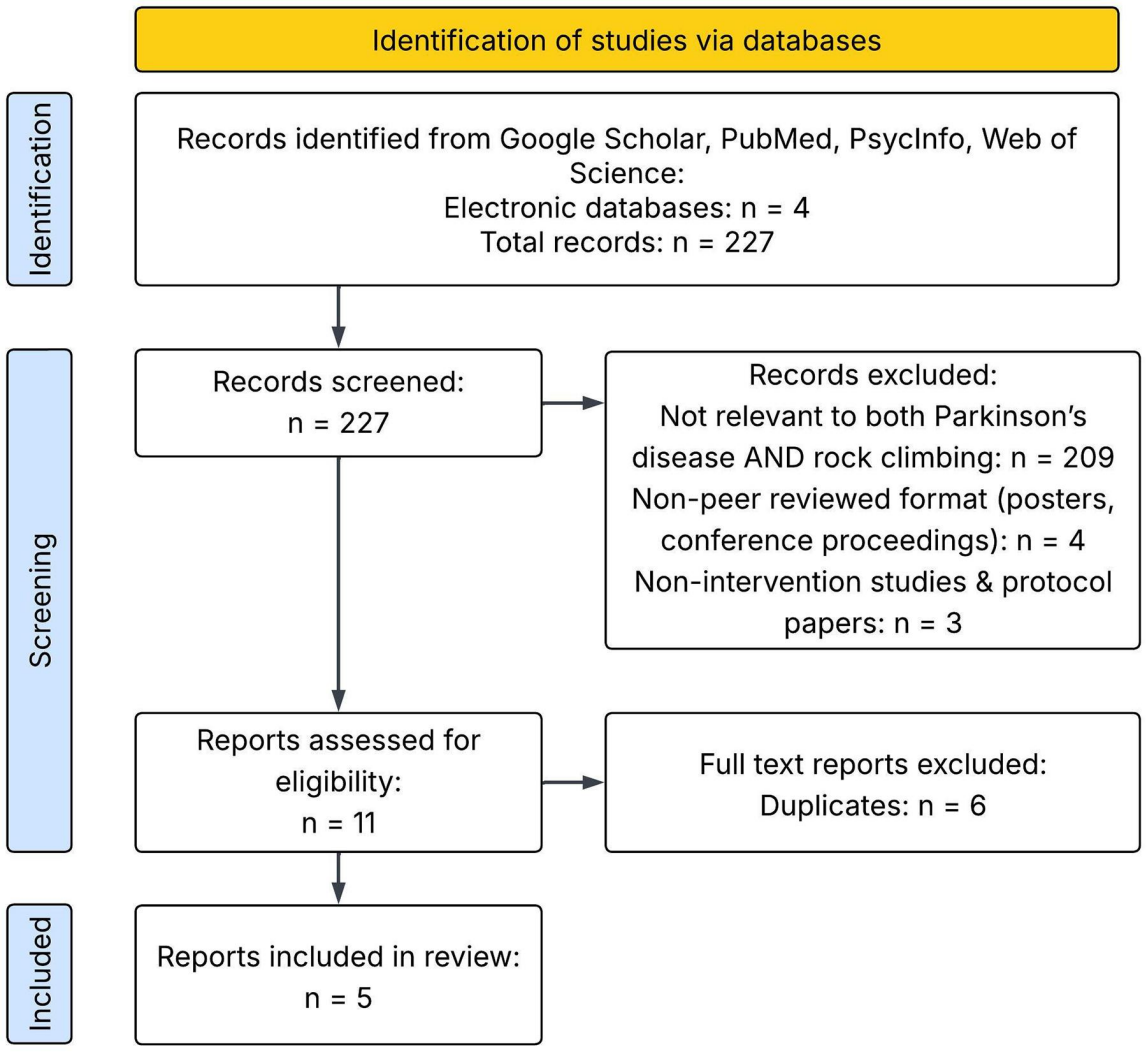
Flow diagram of literature search procedure.

The systematic search of four databases yielded 225 records, of which five met all criteria and were included in this systematic review. The rationale for the exclusion of records is presented in Figure 2.

### Publication quality

Total scores for the quality of each included publication, as well as a summary of results, are presented in Table 1. Quality scores for each item of the assessment tool are available in Table 2. Publication quality scores ranged from seven to 19 with a mean of 15.80 ± 5.22 SD out of 20 points. Intervention description, reporting of results, and definition of dependent variables were the highest-rated items (1.80 ± 0.45 SD) and use of a control group and group randomization were the lowest rated items (both 1.20 ± 1.10 SD).

**Table 1:** Summary of included literature.

Publication	Research Design	Group Assignment	Climbing Intervention FITT	Quality Score (/20)	Assessments	Motor Results Summary^a^
Gassner et al., 2023 (30)	Single-group feasibility trial	Bouldering *n* = 26	12–20 sessions over 4 wk (3–5 25-minute sessions per wk)	7	original survey (post only), adherence rate, 10 m walk and/or 2-min walk, FGA, TAT, 9HPT	10 m walk time decreased 1.0 s^b^ (*p* < .01; *n* = 16; ES not reported). 2-min walk distance increased 27.5 m^b^ (*p* < .01; *n* = 19; ES not reported). 9HPT time decreased left 2.4 s^b^, right 3.4 s^b^, (both *p* < .01; *n* = 15; ES not reported). FGA increased 2.0 points^b^ (*p* < .01; *n* = 15; ES not reported). TAT n.s.; ES not reported (*n* = 8).
Langer et al., 2021 (32)	Semi-blind RCT	SC *n* = 22, UT *n* = 24	12 wk, 90 min/wk	19	MDS-UPDRS-III, original feasibility survey	SC greater improvement than UT in MDS-UPDRS-III total (*p* < 0.001; ES not reported), bradykinesia (*p* = 0.003; ES not reported), rigidity (*p* = 0.016; ES not reported), tremor (*p* = 0.001; ES not reported). Total: SC −12.9; 95%CI [−15.9, −9.8], UT −3.0 L; 95%CI [−6.0, 0.1]. Bradykinesia: SC −5.2; 95%CI [−6.8, −3.6], UT −1.8; 95%CI [−4.0, 0.4]. Rigidity: SC −1.8; 95%CI [−2.6, −1.0], UT −0.3; 95%CI [−0.6, 1.1]. Tremor: SC −4.9; 95%CI [−7.0, −2.8], UT −1.0; 95%CI [−2.5, 0.4].
Langer et al., 2023 (33)	Semi-blind RCT	SC *n* = 22, UT *n* = 24	12 wk, 90 min/wk	19	C7SVA	Significant improvement in C7SVA in SC [−1.7 cm; 95%CI (−2.6, −0.8); ES not reported], not in UT [−0.5 cm; 95%CI (−1.3, 0.2); ES not reported]
Langer et al., 2024 (31)	Semi-blind RCT	SC *n* = 22, UT *n* = 24	12 wk, 90 min/wk	19	IMU-derived kinematic evaluation of normal walk, fast walk, 5StS, TUG, motor dual-task walk, cognitive dual-task walk	Normal walk speed: SC increased [0.09 m/s; 95% CI (0.04, 0.14), *p* = 0.003; ES not reported], UT n.s. Normal walk STA: SC decreased [−0.02 s 95% CI (−0.03, 0.01), *p* = 0.008; ES not reported], UT n.s. 5StS: SC decreased [−2.5 s; 95% CI (−4.43, −0.57), *p* = 0.014; ES not reported], UT n.s. SC cognitive dual-task walk time decreased [12 s; 95% CI (−21.9, −2.4), *p* = 0.017; ES not reported]. TUG time n.s; ES not reported.
Ries et al., 2025 (29)	Quasi-experimental pilot study	SC *n* = 28	12 wk, 24 sessions, 2–3 sessions/ wk	15	ATT, modified CBMS, GS, UE-React, 9HPT	Modified CBMS increased (mean ± SD unit, Cohen's *d*) 6.250 ± 6.942 points, *d* = 0.573. ATT time decreased 2.419 ± 3.417 s, *d* = 0.462. 9HPT time decreased 2.148 ± 3.125 s, *d* = 0.480. UE-React time decreased 0.740 ± 0.998, *d* *=* 0.329. GS n.s.; ES not reported.

ATT, agility t-test; CBMS, community balance and mobility scale; C7SVA, 7th cervical vertebra sagittal vertical axis; ES, effect size; FGA, functional gait assessment; FITT, frequency, intensity, time, type; GS, grip strength; IMU, inertial measurement unit; m, meter; MDS-UPDRS-III, part III of the Movement Disorder Society-sponsored revision of the Unified Parkinson's disease rating scale; min, minute; n.s., not significant; SC, sport climbing; SD, standard deviation; s, seconds; STA, step time asymmetry; TAT, Tinetti assessment tool; TUG, timed up and go; UE-React, upper extremity reaction time test with BlazePods; UT,  unsupervised training; wk, week; 5StS, 5-time sit-to-stand; 9HPT, Nine-Hole Peg Test.

^a^ Selected results motor assessments reported as changes pre-intervention to post-intervention.

^b^ Change score variability unavailable.

**Table 2:** Publication quality evaluation by item.

Publication	Inclusion Criteria	Control Group	Group Assignment	Intervention Description	Dependent Variables	Practical Assessments	Training Duration	Appropriate Tests	Results	Conclusions
Gassner et al., 2023 (30)	0	0	0	1	1	1	0	0	2	2
Langer et al., 2021 (32)	2	2	2	2	2	1	2	2	2	2
Langer et al., 2023 (33)	2	2	2	2	2	2	2	2	1	2
Langer et al., 2024 (31)	2	2	2	2	2	2	2	2	2	1
Ries et al., 2025 (29)	2	0	0	2	2	2	2	2	2	1
Mean ± SD	1.60 ± 0.89	1.2 ± 1.10	1.20 ± 1.10	1.80 ± 0.45	1.80 ± 0.45	1.60 ± 0.55	1.60 ± 0.90	1.60 ± 0.90	1.80 ± 0.45	1.60 ± 0.55

### Study and sample characteristics

The five included articles reflect data from one semi-blind randomized controlled trial, one single-group feasibility trial, and one quasi-experimental pilot study. Three distinct articles were published based on the randomized controlled trial—the article for the primary outcome and two planned secondary analyses of additional outcomes. Two studies were conducted in Austria, and one was conducted in the United States of America. Two of the climbing interventions were top-rope SC and were conducted in commercial climbing gyms. One intervention involved bouldering and was conducted in an inpatient neurorehabilitation center.

Participant characteristics are presented in Table 3 and study details are presented in Tables 1, 3. A total of 106 participants were consented and enrolled, 102 of whom are included in the demographic analysis. Two participants were excluded from further analysis due to study dropout, leaving a total sample of 100 distinct participants. No participants had former climbing experience. All three studies included more male than female participants, and the composite sample was 68% male. The age range for the composite sample was 45–78 years.

**Table 3:** Study and sample characteristics.

Study	Country & Setting	*n* Consented	Sex	Age (years)	Disease Duration (years)	H&Y Stage	Participants Excluded from Analysis
Gassner et al., 2023 (30)	Austria, inpatient neuro rehabilitation center	26	Female: 7 Male: 19	68 (55–78)	7 (1–23)	1: 4 (16%) 2: 17 (68%) 3: 4 (16%)	0
Langer et al., 2021 (32), 2023 (33), 2024 (31)	Austria, commercial climbing gym	SC: 24^a^ UT: 24	Female: 18 Male: 30	SC: 65 (45–78) UT: 64 (49–78)	SC: 6 (<1–12) UT: 5 (<1–15)	SC 2: 20 (83%) SC 3: 4 (17%) UT 2: 22 (92%) UT 3: 2 (8%)	SC: 2, UT: 0. These participants were included in reported demographics and individual characteristics were not reported. Reason for exclusion: Dropout (non-PD medical) *n* = 1, dropout (personal) *n* = 1
Ries et al., 2025 (29)	USA, commercial climbing gym	32^b^	Female: 8 Male: 20	66 (45–78)	4 (1–13)	1: 18 (64%) 2: 7 (25%) 3: 3 (11%)	4. Reason for exclusion: Dropout (lack of interest) *n* = 2, dropout (scheduling) *n* = 1, dropout (unrelated injury) *n* = 1
Total		106	Female: 33 (32%) Male: 69 (68%)	45–78	1–23	1: 22 (22%) 2: 66 (65%) 3: 13 (13%)	6

Three articles by Langer were published from the same sample and the characteristics are therefore presented together. Age and disease duration are presented as Mean (Range) in years. H&Y, Hoehn and Hahr; *n*, number of participants; SC, sport climbing; UT, unsupervised training; USA, Unites States of America.

^a^ Two participants who dropped out were included in the source article demographic analysis and this aggregate sample demographic analysis.

^b^ Four participants who dropped out were not included in the source article demographic analysis or this aggregate sample demographic analysis.

Time since PD diagnosis ranged from two months to 23 years. Study-level averages of disease durations ranged from four to seven years. Ries et al. (29) and Gassner et al. (30) included participants from Hoehn and Yahr (H&Y) stages one through three. Langer et al. (31–33) included only participants in H&Y stages two and three. In the composite sample, 22% were in H&Y stage one, 65% were in H&Y stage two, and 13% were in H&Y stage three. Individuals with H&Y scores of four or greater were not eligible for any of the included publications.

The randomized controlled trial compared top-rope SC to unsupervised exercise training (UT) (31–33). Participants in the SC group completed 90 min of SC per week for 12 weeks. Climbing sessions were facilitated by an instructor in groups of three to four participants. The instructor was the primary belayer, and participants were permitted to belay one another after receiving approval from the instructor. Participants in the UT group were educated about and instructed to follow PD-specific exercise recommendations. The recommendation involved 150 min of moderate or 75 min of vigorous aerobic physical activity per week, resistance training two times per week, and balance training three times per week (32, 34).

The single-group feasibility trial was conducted in an inpatient rehabilitation center (30). Participants completed three to five bouldering sessions per week for four weeks under the supervision of a physiotherapist. The number of total sessions for each participant ranged from 12 to 20 with an average of 16. Bouldering sessions occurred in groups of two participants, and each session was 25 min in duration.

The quasi-experimental pilot study involved top-rope SC at a commercial climbing gym (29). Participants completed 24 SC sessions, with no more than three sessions per week. Program volunteers supervised and belayed participants. The duration of each session was not included in the source publication.

### Intervention feasibility and acceptability

All studies tracked adverse events and climbing-related injuries, and all reported zero. This indicates preliminary feasibility. Across the three studies, 76 of 82 (92.6%) of participants in climbing-based intervention groups completed pretests, climbing intervention, and post-tests. The reasons for exclusion and dropout are presented in Table 3. Authors of two publications further assessed feasibility using participation and dropout rates (30, 32). Attendance to exercise sessions was greater than 99% in both studies. Participants in the study by Langer et al. (32) missed a combined three out of 264 sessions and participants in the study by Gassner et al. (30) missed zero out of 416 sessions. Acceptability was assessed through surveys evaluating participant desire to continue climbing and to promote the activity to a peer. Of the participants in Gassner et al., 96% would recommend climbing to a peer and 70% reported motivation to continue climbing (30). Similarly, Langer et al. found that 48% of participants in the SC group continued to climb 12 months after the end of the intervention. An additional 36% expressed a desire to continue climbing but experienced logistical and health barriers. Furthermore, 25% of participants in the comparator UT group began climbing when presented with the opportunity upon conclusion of the study (32).

### Motor symptoms

#### MDS-UPDRS-III

One research group utilized part III of the Movement Disorder Society-sponsored revision of the Unified Parkinson's Disease Rating Scale (MDS-UPDRS) to compare the effects of SC and UT (32). The SC group exhibited an average change in total MDS-UPDRS-III score of −12.9 points [95% CI (–15.9, −9.8)] over 12 weeks, compared to −3.0 points [95% CI (–6.0, 0.1)] in the UT group (32). The SC group demonstrated significantly greater improvement than the UT group in each of the MDS-UPDRS-III sub-domains—tremor [SC: −4.9 95% CI (–7.0, −2.8), UT −1.0, 95% CI (–2.5, 0.4), *p* = 0.001], rigidity [SC: −1.8, 95% CI (–2.6, −1.0), *p* = 0.016, UT −0.3, 95% CI (–0.6, 1.1), *p* = 0.003], and bradykinesia [SC: −5.2, 95% CI (–6.6, −3.6), UT −1.8, 95% CI (–4.0, 0.4)] (32).

#### Locomotion

A summary of locomotor performance and spatiotemporal parameters of gait are presented in Table 4. In general, gait speed on simple and complex tasks increased following climbing interventions. Participants completed distance-based tasks more quickly and walked farther in time-based paradigms (29–31). For example, Langer et al. (31) found that participants in the SC group walked 0.09 m/s faster on a 20-meter normal-speed walking task. Similarly, participants in the bouldering study by Gassner et al. (30) completed a 10-meter walk one second faster, which is an increase in gait speed of 0.10 m/s. In the article by Langer et al. (31), gait speed tended to increase by between 0.05 and 0.10 m/s for other test paradigms in the SC group and not change significantly in the UT group (see Table 4).

**Table 4:** Summary of locomotor outcomes.

Publication	Locomotor Paradigm	Outcome Parameter	Results
Gassner et al., 2023 (30)	10-meter walk	Completion time^a^	Pre: 7.5 s 95% CI [1.1, 13.9], Post: 6.5 s 95% CI [0.1, 12.9], *p* < .01
	2-minute walk	Total distance^a^	Pre: 149.5 m 95% CI [−111.0, 410.0], Post: 177.0 m 95% CI [−140.7–494.7], *p* < .01
	Functional Gait Assessment	Observer-rated ordinal scale, range 0–30^a^	Pre: 26.0 95% CI [−24.8, 76.8], Post: 28.0 95% CI [2.6, 53.4], *p* < .01
Langer et al., 2024 (31)	20-meter walk, normal pace^b^	Gait speed^a^	SC: mean difference (post – pre) 0.09 m/s 95% CI [0.04, 0.14], *p* = 0.003. UT: –0.01 m/s 95% CI [–0.07, 0.05] *p* = 0.011
		Step time asymmetry^a^	SC: mean difference –0.02 s 95% CI [− 0.03, 0.01], *p* = 0.008. UT: ns
	20-meter walk, fast pace^b^	Gait speed^a^	SC: mean difference 0.1 m/s 95% CI [0.06, 0.21], *p* < 0.001. UT: mean difference 0.07 m/s 95% CI [–0.01, 0.15] *p* = ns
		Step time^a^	SC: mean difference –0.02 s 95% CI [−0.01, −0.03], *p* = 0.002. UT: ns
		Stride time^a^	SC: mean difference –0.05 s 95% CI [−0.02, −0.07], *p* = 0.002. UT: ns
		Stance time^a^	SC: mean difference –0.03 s 95% CI [−0.01,−0.05], *p* = 0.004. UT: ns
	Motor dual-task walk^b^	Gait speed^a^	SC: mean difference 0.05 m/s 95% CI [0.05, 0.15], *p* = not reported. UT: mean difference 0.04 m/s 95% CI [–0.02, 0.1] *p* = ns
	Cognitive dual-task walk^b^	Gait speed	SC: mean difference 0.06 m/s 95% CI [–0.04, 0.16], *p* < 0.001. UT: mean difference 0.01 m/s 95% CI [–0.08, 0.10], *p* = ns
	3-meter TUG^b^	Gait speed	SC: mean difference 0.04 m/s 95% CI [0.01, 0.08], *p* = ns. UT: 0.00 m/s 95% CI [–0.04, 0.03], *p* = ns
		Step time^a^	SC: mean difference –0.02 s 95% CI [–0.29, –0.04], *p* = 0.011. UT: ns
		Stride time^a^	SC: mean difference –0.40 s 95% CI [–0.60, –0.10], *p* = 0.008. UT: ns
		Stance time^a^	SC: mean difference –0.30 s 95% CI [–0.48, –0.06], *p* = 0.013. UT: ns
		Swing time^a^	SC: mean difference –0.10 s 95% CI [–0.11, –0.01], *p* = 0.017. UT: ns
		Double limb support time^a^	SC: mean difference –0.20 s 95% CI [–0.19, –0.02], *p* = 0.019. UT: ns
	7-meter TUG^b^	Gait speed^a^	SC: mean difference 0.09 m/s 95% CI [0.02, 0.15], *p* = 0.011. UT: 0.03 m/s 95% CI [–0.004, 0.07], *p* = ns
		Steps/m (called cadence)^a^	SC: ns. UT: mean difference –0.7 steps 95% CI [–1.26, –0.08], *p* = 0.027.
		Step time^a^	SC: ns. UT: mean difference 0.10 s 95% CI [0.02, 0.21], *p* = 0.015
		Stride time^a^	SC: ns. UT: mean difference 0.20 s 95% CI [0.04, 0.42], *p* = 0.022
		Stance time^a^	SC: ns. UT: mean difference 0.20 s 95% CI [0.03, 0.34], *p* = 0.019
		Double limb support time^a^	SC: ns. UT: mean difference 0.10 s 95% CI [0.01, 0.14], *p* = 0.026
	Dual-task TUG	Gait speed	SC: mean difference 0.04 m/s 95% CI [0.001, 0.07], *p* = ns. UT: mean difference –0.01 m/s 95% CI [–0.05, 0.03], *p* = ns
	ISAW^b^	Gait speed^a^	SC: mean difference 0.12 m/s 95% CI [0.07, 0.18], *p* < 0.001. UT: mean difference 0.00 m/s 95% CI [–0.07, 0.06], *p* = ns
		x	x
Ries et al., 2025 (29)	Agility T-Test	Completion time^a^	Mean difference −2.419 ± 3.417 s, *p* < 0.001, Cohen's *d* = 0.462 (95% CI 0.078, 0.838)

ISAW, instrumented stand and walk test; ns, not significant. SC, sport climbing; TUG, timed up-and-go; UT, unsupervised training. The IMU-derived parameters of interest for all paradigms in Langer et al. (21) were gait speed (m/s), cadence (steps/m), time (s), stride time (s), stance time (s), swing time (s), double support time (s), double limb support variability, and asymmetry (s). Only significant IMU-derived results are reported in this table, and full results are available in the Supplementary Materials of the original publication. In the article by Langer et al. (31), the cognitive dual task was a 20-meter walk and serial subtraction by seven or three, and the motor dual task was a 20-meter walk and marking boxes on paper. The cognitive task during the dual-task TUG was serial subtraction by three from 202. Apparent contradictions between confidence intervals and means are reported here as in the source publication and associated Supplementary Materials.

^a^ Indicates significant differences between pre-test and post-test in at least one condition.

^b^ Indicates that a paradigm was instrumented with IMUs.

Langer et al. (31) instrumented several locomotor tasks using inertial measurement units (IMUs). The duration of some gait cycle phases decreased significantly in the SC group but not the UT group. Gait phase parameters, however, are presented in absolute time rather than relative duration in the gait cycle, and it is therefore unclear if the spatiotemporal pattern changed or if significant changes in cycle length are only due to increased speed. Notably, Langer et al. (31) reported a variable labeled cadence; however, it is recorded in steps per meter, not steps per minute. This variable is therefore a measure of step length and none of the included articles examined the effects of climbing interventions on cadence.

#### Manual dexterity

Two research groups evaluated manual dexterity using the 9-hole peg test, in which a faster time indicates better dexterity (29, 30). Ries et al. (29) found a decrease in task completion time of 2.148 s (SD 3.125, *p* *<* *0.001, d* = 0.480). Similarly, Gassner et al. (30) found a decrease of 2.4 s with the left hand (*p* = 0.003) and 3.4 s with the right hand (*p* = 0.001). The changes in performance in both studies represent improvements of approximately 10%.

#### Balance and posture

Ries et al. assessed the effects of a climbing intervention on a truncated version of the Community Balance and Mobility Scale (29). Participants demonstrated a statistically significant improvement with a mean increase of 6.250 out of 60 points (SD = 6.942, *p* < 0.001, *d* *=* 0.573).

Langer et al. evaluated the effects of SC and UT on participants’ static posture by measuring the seventh cervical sagittal vertical axis (C7SVA), the distance between the 7th cervical vertebra and a wall against which the participant stood. Participants in the SC group demonstrated a significant decrease in C7SVA distance [mean change −1.7 cm; 95% CI (−2.6, −0.8)], whereas the UT group did not [mean change −0.5 cm; 95% CI (−1.3, 0.2)]. Additionally, a regression model found a significant association between change in C7SVA distance and assignment to the SC group, including when controlling for age and anthropometric characteristics (coeff. 1.2; *p* = 0.048) (33).

#### Reaction time

Ries et al. evaluated reaction time by measuring the total time to tap BlazePod® contact sensors as they illuminated in a random sequence 30 times. Participants performed the test an average of 0.740 s faster following 12 weeks of SC (SD = 0.998, *p* < 0.001, *d* = 0.329 (29).

#### Strength

Two groups assessed the effects of climbing interventions on muscular strength. Langer et al. assessed lower extremity strength using the time to perform the five-time sit-to-stand (5StS) test (31). Participants completed the 5StS in a significantly shorter time after the climbing intervention [mean difference = −2.5 s, 95% CI (−4.43, −0.57), *p* = 0.014]. Conversely, Ries et al. evaluated grip strength using a handheld dynamometer and did not find a statistically significant difference in force between the pre- and post-intervention timepoints (mean difference 1.661 ± 14.337 lbs., *p* = 0.273) (29).

### Non-motor symptoms

In general, the included publications reported few pre-post assessments of the non-motor symptoms of PD. Gassner et al. used an original survey to evaluate participant perceptions of the effects of climbing on quality of life and several PD-associated non-motor symptoms. Over 50% of respondents reported improved quality of life, increased motivation, increased self-awareness, and decreased fatigue (30). These results, however, are based on self-report after the intervention only.

Additionally, the Langer group collected data related to non-motor symptoms and quality of life, but results are scantly reported. Baseline and endpoint data related to quality of life, mood, and fatigue were included as covariates in a regression model and were found to not be associated with improved posture. However, no quantitative data were provided describing changes in the Parkinson's Disease Questionnaire, Beck Depression Inventory, Parkinson's Fatigue Scale, Physical Activity Scale for the Elderly, or Falls Self-Efficacy Scale.

#### Cognition

Subjectively, 70% of participants in the study by Gassner et al. rated their concentration as “improved” on a post-intervention questionnaire. Three publications from one study reported the results of the MMSE at baseline as a sample characteristic; however, post-intervention results were not reported (31–33). The mean MMSE score was 29.3 ± 0.2 (SEM) (32). Participants in Langer et al. (31) completed ten subtractions of the serial sevens task as quickly as possible. Participants in the SC group completed the task an average of 12 s faster following the intervention [95% CI (−21.9, −2.4), *p* = 0.017], compared to five seconds faster in the UT group [95% CI (−6.5, −3.0), *p* < 0.001]. Participants also completed two cognitive-motor dual task paradigms—serial subtraction of threes from 202 simultaneously with an instrumented 20-meter walk test and a 3-meter timed up-and-go (TUG) test. Participants in both the SC and UT groups demonstrated decreased gait speed on the dual-task 20-meter walk compared to the single-task 20-meter walk. The SC group demonstrated a greater reduction in relative speed after the climbing interventions (SC: pre −0.4 m/s, post −0.5 m/s; UT: pre −0.4 m/s, post −0.4 m/s). Walking speed on the 3-m TUG was 0.1 m/s slower under dual-task conditions than single-task conditions for all groups and timepoints (31).

## Discussion

The purpose of this systematic review was to synthesize and evaluate published research that examined climbing-based interventions for PD. Five publications based on three studies met the inclusion criteria. All of the included articles have been published since 2021. Although the current evidence base is small, the existence of recent publications conducted by groups on multiple continents suggests that therapeutic climbing for PD is an emerging area of inquiry. Furthermore, climbing may satisfy exercise recommendations for people with PD in some or all of the four recommended domains—aerobic exercise, resistance training, flexibility training, and neuromotor exercise (13).

### Search strategy and record inclusion

The broad search phrase “Parkinson* AND sport climb*” was selected to minimize the omission of potentially relevant records while initially screening out studies unrelated to therapeutic climbing. Therapeutic climbing can include multiple disciplines such as SC and bouldering that vary by level of inherent risk (see Figure 1). Sport climbing refers to a discipline of climbing in which a rope arrests falls. Top-rope climbing is a sub-discipline of SC in which the rope passes through an anchor point that is always above the climber, thus minimizing the amount of slack in the system and the distance a climber can fall before being caught by the belayer. Bouldering is a distinct discipline of climbing on low walls without a rope, and ground pads are used to reduce the impact of a fall. The search phrase “sport climb*” was selected because the authors anticipated that SC, especially top-rope, would be the principal intervention type, because fall risks are highly mitigated. However, even when using the “sport climb” search term, the search returned one record for which the intervention was bouldering (30). This article met all inclusion criteria because bouldering is a climbing-based intervention. Furthermore, no exclusion criteria were established, so the article by Gassner et al. was included in this review. The database searches and reference chaining revealed no additional bouldering interventions that met the inclusion criteria of this review.

The search returned multiple records that did not meet the inclusion criteria, but may be useful in informing clinical decision making related to climbing for people with PD. Specifically, the search returned conference posters or presentations by Woolstenhulme et al. (35) and Reis et al. (36) that were not peer reviewed and were therefore did not meet the inclusion criteria for this review.

### Safety, feasibility, and acceptability

Despite its common perception as an extreme sport, climbing, especially top-rope SC, is a safe form of exercise and therapeutic modality for individuals of various ages, including people with neurological, orthopedic, and psychiatric diagnoses (21). The lack of climbing-related injuries and adverse events in the included literature suggests that climbing may be a safe and feasible form of exercise for people with PD, especially in the mild and moderate stages of the disease. However, none of the eligible publications included participants with symptoms more advanced than H&Y stage three, and the feasibility of climbing for people with advanced disease progression is not addressed in the current literature. The inclusion of only participants in the early and moderate stages of disease progression is consistent with reviews of other balance-intensive exercise types such as boxing and Tai Chi (37, 38). These balance-intensive forms of exercise may therefore be contraindicated or not consistent with clinical priorities or clinician discretion for individuals in the later stages of PD progression.

Bouldering results in approximately five times as many injuries per 1000 h compared to top-rope climbing (23). This disparity in risk may be especially notable for older populations prone to fall-related injuries. The safety and feasibility of bouldering for people with PD was assessed in one article (30). No injuries or adverse events occurred, suggesting preliminary feasibility. The climbing intervention, however, differed from typical bouldering done as exercise in climbing gyms. Modifications were made to the wall height and support was provided to minimize both the probability and consequence of falls. The wall used in the study was two meters high, whereas bouldering walls in commercial climbing gyms are typically three to four meters high (39). Additionally, participants in the bouldering study by Gassner et al. (30) received support from physiotherapists, further minimizing fall risk. Therefore, the feasibility of bouldering as a form of exercise independent from physiotherapy for people with PD remains unexamined. Researchers may consider conducting further investigation into the feasibility and effectiveness of bouldering because bouldering and mixed bouldering/top-rope facilities are far more prevalent than top-rope only options, and the independence of bouldering allows for increased flexibility in exercise scheduling (40).

With respect to acceptability, 92.6% of participants in climbing intervention groups across all studies completed the training period and post-intervention testing (29, 30, 32). Furthermore, participants who completed climbing interventions attended over 95% of sessions (30, 32). Participant retention and adherence to exercise in people with PD is highly variable, ranging from 60% to 100% across exercise types (41). The relatively high retention and adherence suggest that climbing-based interventions may be comparably acceptable to, or perhaps more acceptable than, other exercise types for people with PD. Additionally, 70% and 84% of participants across two studies expressed interest in continuing climbing as exercise following the end of the study period, providing further evidence of high acceptability (30, 32).

Climbing may be an acceptable type of exercise because it is consistent with numerous predictors of exercise adherence in older adults and people with PD. These factors include programs tailored to one's abilities, social and professional support, experiences of mastery, and center-based programs (42, 43). The specialization of facilities and materials, especially for SC, means that climbing is largely a center-based activity. Additionally, SC requires a peer, volunteer, therapist, or exercise interventionist to belay the climber, thus facilitating social interactions and social support (21, 44). The beneficial social nature of climbing is supported in the included literature, as Gassner et al. found that 67% of participants reported enjoying the social aspect of the climbing intervention (30). Indoor climbing centers include surfaces of different angles and create, or set, routes of varying levels of difficulty (45). This variation allows for tailored experiences for each participant, facilitating appropriate challenges while allowing for individual accomplishment and mastery (44, 46). In general, the physical and psychosocial characteristics of climbing as exercise may plausibly facilitate exercise retention and adherence.

The level of participant activity, such as time spent climbing, rating of perceived exertion, or repetitions, were not reported in the included literature. Future researchers should increase the reporting detail of exercise sessions and assess adherence using parameters other than attendance for people with PD.

### Motor outcomes and proposed mechanisms

Improvements in the cardinal motor symptoms of PD were observed through a reduction of MDS-UPDRS-III scores. Participants’ scores decreased by an average of 12.9 points, which exceeds the minimal clinically important difference of 3.25 points (32, 47). Notably, none of the included articles examined the effects of bouldering on MDS-UPDRS-III scores. Despite the limitations of the MDS-UPDRS, it is currently considered a gold-standard assessment and its absence in the bouldering literature is indicative of the difference in the strength of the evidence between climbing disciplines. Motor symptom improvement was especially notable in the bradykinesia and tremor sub-scales. Bradykinesia severity is associated with poor quality of life, suggesting that people with PD may experience clinically meaningful changes outside of the exercise setting (48). The results of the study by Langer et al. (32) are consistent with a previous meta-analysis that indicates that resistance exercise ameliorated motor symptoms on the MDS-UPDRS more than a passive control (49). The similarities in movement characteristics and task demands suggest that climbing may plausibly improve motor symptoms through the same mechanisms as resistance training with machines, free-weights, body weight, or resistance bands, though no direct evidence of this phenomenon existed prior to the publication of the included studies.

Two publications examined the effects of climbing on measures of muscular strength, one using the 5StS and another using a hand dynamometer (29, 31). Participants demonstrated improvements in 5StS time but not grip strength. Although grip strength is a predictor of climbing performance, beginner climbers rely more on balance, technique, and lower extremity strength to complete climbing routes (22, 50). Therefore, participants may not have worked the finger and forearm flexors at an intensity that would lead to strength gains. Improvements on the 5StS test suggest improved lower extremity strength; however, the test paradigm has several limitations. The 5StS requires balance as well as strength, and indicators of balance improved following climbing, potentially confounding the results (29). Furthermore, time to perform five repetitions is not a pure measure of strength. Future researchers may use one-repetition maxima or isometric force tests of various major muscle groups to evaluate muscular strength changes following exercise interventions. Such results may be used to compare the mechanisms of climbing and resistance exercise.

In a previous study comparing exercise types, people with PD demonstrated improved muscular strength following resistance exercise but not aerobic exercise (51). Similar demands and initial results demonstrating improvement on the 5StS suggest that climbing and resistance training may have mechanistic similarities. Both climbing and resistance training involve repeated concentric and isometric contractions, sometimes working muscles to failure. Both exercise types also contribute to acute fatigue and long-term adaptation in major muscle groups (20, 22). Resistance training that involves working to failure may contribute to greater improvements in motor symptoms than other exercise types (10, 52, 53). Climbing inherently promotes working to failure as it is goal-oriented, with climbers regularly performing multiple attempts to failure while working towards mastery of a specific route. The physiological and conceptual mechanisms underlying the motor benefits of resistance exercise, and perhaps climbing, include strengthening muscles, improving motor learning, regulating corticospinal excitability, regulating gene expression, altering mitochondrial function, and promoting neuroplasticity (49, 54). None of these mechanisms, however, have been directly examined in the context of climbing-based interventions for PD.

Quantitative evaluation of locomotor tasks indicated that people with PD walked faster after SC or bouldering interventions (29–31). These results are consistent with improvements in mobility with various other exercise types—especially aerobic and neuromotor exercise such as walking, dance, Tai Chi, and yoga (17). This suggests that climbing may engage similar mechanisms as aerobic and neuromotor exercise.

Heart rate scales with effort during climbing, suggesting that climbing may be an aerobic stimulus (20). In healthy recreational climbers, moderate-to-hard SC results in heart rates at or above the suggested range of high-intensity exercise for people with PD—80% to 85% of maximum [heart rate peak 83 ± 8% SD (males); 90 ± 5% SD (females)] (13, 55). Climbing may therefore engage similar mechanisms to conventional aerobic exercise types, such as walking and cycling. However, climbing sessions consist of intervals of vigorous activity and rest, whereas sustained aerobic efforts may be better than interval training for ameliorating the symptoms of PD (56). Differences may exist in how SC and bouldering affect heart rate, as bouldering is performed for shorter, more intense intervals, while SC involves longer, more sustained efforts. However, none of the included studies measured participants’ heart rates during either bouldering or SC, leading to a current lack of empirical evidence of this effect. Multiple molecular and neural network mechanisms are hypothesized to contribute to the effects of aerobic exercise for people with PD. These mechanisms include regulation of neurotrophic factors, increased neuroplasticity, reduced neuroinflammation, modulation of corticomotor excitability, and regulation of signaling pathways associated with autophagy and apoptosis 10). Aerobic exercise may therefore be neuroprotective and is associated with favorable outcomes in the motor and non-motor domains (17, 57, 58). Future climbing and aerobic exercise researchers may assess biomarkers to compare the effects of exercise types at the molecular level.

Changes in motor performance may also, perhaps, be attributed to task and mechanistic similarities to established forms of neuromotor exercise. Neuromotor exercise involves training that targets dual-task performance, balance, posture, and agility (13). Practices such as yoga, Tai Chi, and Qigong often integrate elements of neuromotor training (10, 13). Mind-body practices may promote awareness of sensation and motor performance. Both attention and improved sensorimotor integration may be mechanisms underlying the benefits of neuromotor exercise for people with PD (10). Climbing may promote a similar awareness because the limited availability of holds necessitates multi-step motor planning and precise proprioceptive awareness (21, 59). Balance exercise is a form of neuromotor exercise that aims to improve responses to everyday perturbations by introducing destabilizations and systematically training stability on a smaller base of support (10, 60). Balance training is associated with decreased risk of falls in people with PD (61). The neurophysiological mechanisms underlying balance training may involve improving sensory integration, improving cognitive-motor dual tasking, and developing compensatory strategies (10). Balance exercise is naturally integrated into climbing because the availability and size of holds limit the base of support and moving one's feet requires unweighting a limb, which is a form of balance destabilization (59). Observed improvements in reaction time, balance, and posture in the included literature may suggest mechanistic similarities between climbing and established forms of neuromotor exercise (61).

Results related to the neuromotor effects of climbing, however, are mixed. For example, there were no significant changes in gait parameters during dual-task walking (31), which is associated with falls in people with PD (62). However, participants demonstrated improved performance on a functional balance assessment and more upright posture following a SC intervention (29, 33). Future research may examine the effects of climbing on balance and postural changes associated with fall risk using validated surveys and test paradigms.

Flexibility training, which is often integrated into warmup and cooldown phases of exercise sessions, is important for reducing PD-associated rigidity (13). Apart from acute changes in mobility, the effects and mechanisms of flexibility training in PD have not been thoroughly examined. Flexibility is a predictor of climbing performance, and various common climbing movements require large ranges of motion (50). This suggests face validity of changes in flexibility following climbing. None of the publications included in this review, however, quantitatively evaluated the effects of climbing on flexibility.

### Non-motor outcomes and proposed mechanisms

One included article evaluated participant perception of the effects of climbing on PD-associated non-motor symptoms and phenomena (30). Participants reported perceived improvements in fatigue, self-confidence, concentration, motivation, courage, sociability, and other psychosocial phenomena. The survey, however, was only given after the intervention and explicitly asked about improvements. The results are therefore prone to self-report bias and the observer-expectancy effect. The Langer et al. group collected validated measures of non-motor symptoms including the MMSE, Beck Depression Inventory, Parkinson's Disease Questionnaire, and Parkinson's Fatigue Scale (31–33). However, it is unclear if these questionnaires were repeated after the intervention, as no pre-post comparisons were returned in the search for this review.

One research group evaluated cognition using the serial sevens task and a serial threes/walking dual task (31). A decrease of an average of 12 s for 10 subtractions in the serial sevens task suggests improved working memory and attention following the climbing intervention. This single result, however, reflects low certainty and should be interpreted with caution.

Previous researchers have found improved cognition in people with PD following non-climbing exercise interventions, including both aerobic and resistance exercise (13, 49, 63). The cognitive benefits of exercise may be associated with neurotropic and neurotrophic mechanisms, as evidenced by increased hippocampal volume in healthy older adults following exercise (63). As climbing has similar metabolic characteristics to resistance exercise, these mechanisms may be engaged through climbing, thus warranting robust assessment of cognitive outcomes. Furthermore, social engagement facilitated by exercise may contribute to improved cognitive and psychosocial symptoms (64).

Outside of the cognitive domain, aerobic exercise may ameliorate depressive symptoms and improve patient-rated quality of life more effectively than other exercise types, while and resistance and mixed-type exercise may contribute to decreased autonomic dysfunction and improved sleep for people with PD (57, 63). The cogent links between the characteristics of climbing and other exercise types warrants further investigation on the effects of climbing-based interventions on non-motor symptoms of PD.

Overall, the dearth of pre-post assessment of validated measures of non-motor symptoms is a gap in the current literature. In the cognitive domain, future researchers may consider using validated tools such as the Montreal Cognitive Assessment (MoCA), which would allow for comparison of results across time, as well as with established clinical trials of other exercise modalities for people with PD (65). The MoCA also recently became available as a quick, automatically scored digital version with convergent validity to the paper-based test (66). This test may allow for easier implementation of pre-post assessment in future research. Similarly, future researchers may use validated questionnaires to assess quality of life and non-motor symptoms including changes in sleep, mood, motivation, and autonomic function.

### Climbing-specific mechanisms

Sport climbing and bouldering integrate opportunities to practice functional skills and demanding neuromotor tasks. Non-climbing elements of SC, including belaying and “tying in”, involve coordinated gross and fine motor sequences. Belaying requires a precise sequence of large, coordinated upper extremity movements. Langer et al. (32) permitted participants to belay one another following training and approval from the instructor. Participants who belayed their peers may have been more active during sessions and may have benefited from learning the motor sequence of pulling in and capturing slack. Roped climbing also requires climbers to “tie in” to the belay system using knots that involve complex coordinated movement sequences (22). Tying and untying these knots may have served as fine motor coordinative exercise for participants with PD. None of the included studies reported if participants tied in themselves, were aided by instructors and volunteers, or used an alternative attachment system such as locking carabiners.

As there are no ropes in bouldering, all falls result in ground-falls, and boulderers are trained to roll out of a fall, rather than landing on their feet. While ground-falls present additional risk compared to falls caught by a rope, they may also facilitate practice rising from the ground. Rising from the ground is an important functional skill for people with PD and is often a goal in interdisciplinary therapies such as occupational therapy and physiotherapy (67).

### Research quality

The rigor and quality of reporting in the included literature is variable, ranging from 7 to 19 on an established 20-point scale (26, 27). The use of comparison groups and equal assignment of participants were the lowest scoring categories on the quality assessment. Only Langer et al. included a comparison group (31–33), though the equivalence of the comparison group was limited because center-based SC was compared with self-reported unsupervised exercise. While study participants in the UT group were contacted by phone every 7–10 days and reported weekly exercise levels of approximately twice the World Health Organization's recommended exercise guidelines for PD patients, no technology was used to verify the intensity or frequency of exercise performed (32). This presents a significant issue in comparing the two groups, as participants in studies employing self-report methods often overestimate or underestimate exercise adherence (43). Additionally, the wide varieties of exercise available to participants in the unsupervised training group introduced much more variation in exercise type compared to the SC group. The comparison of SC (an inherently social activity) to unsupervised training (which may be performed either alone or in a group setting) further confounds direct comparison of the two groups. This is notable because social factors such as the presence of an exercise partner have been shown to influence exercise adherence in people with PD (42). Future studies may mitigate this limitation and allow for more direct comparison between groups by measuring exercise adherence through exercise-tracking technologies or by supervising control group participants in one standard form of exercise in a center-based environment.

The study by Gassner et al. (30) has numerous methodological limitations and should therefore be interpreted with caution. First, the training duration was four weeks, which is typically too short for many physiological adaptations to exercise to occur (10). Furthermore, participants completed multiple concurrent physical activity and neuromotor therapies, minimizing the extent to which changes in motor performance may be specifically attributed to bouldering.

In addition to variable quality and research designs, there are several errors and oversights in the publications included in this review. For example, the path and continuity of the rope in a schematic of the top-rope climbing setup in one of the publications is inaccurate (32). Another example of oversight was the reporting of effect size with Cohen's *d* despite the method indicating that Hedge's *g* would be used to account for the small sample size (29). A third case is the absence of quantitative results for nonmotor questionnaires that, per the method, were collected at baseline and post-intervention. Together, these oversights call into question the rigor of manuscript preparation and suggest an increased risk of bias in the current literature. The quality assessment tool used in this review was not sensitive to these publication-level issues, suggesting that the choice of quality assessment tool may be a limitation of this review.

### Methodological considerations

The literature included in this review generally supports the feasibility of scientific inquiry into climbing-based interventions for PD. Data in four of the five publications were collected by evaluators who were blinded to group assignment or intervention participation (30–33). Future researchers, however, should consider feasibility and validity of outcome measurement tools when creating study designs.

Although the MDS-UPDRS-III is a standardized and widely recommended assessment of motor symptoms, it has limited precision and sensitivity to change, especially in the early stages of PD (68). This may limit the sensitivity of future studies, especially those with longitudinal components or involving *de novo* participants. Furthermore, the MDS-UPDRS must be conducted by a trained facilitator, which adds additional barriers to the use of the tool in larger, multi-center effectiveness trials. Furthermore, trained and certified clinicians are not always consistent in their evaluation of motor symptoms using the MDS-UPDRS-III (69). Future researchers may therefore consider alternative validated tools to evaluate the motor effects of climbing interventions. These tools may include digital options which can be used to gather more objective measurements without the use of trained facilitators.

Many of the publications included in this review used wearable sensors and other digital tools to objectively quantify motor performance. Langer et al. used wearable IMU sensors attached to the ankles, wrists, lumbar spine, and chest to analyze participants’ gait (31). The use of IMU sensors for gait analysis is well-validated and increasingly common in the PD literature, thus allowing for comparison to other treatment approaches (70–72). The portability and flexible application of IMUs may also allow future researchers to collect data during climbing.

One included study used BlazePod® illuminating contact sensors to evaluate reaction time and agility (29). These sensors allowed the researchers to control testing paradigms and collect precise performance times. The use of random sequences, however, may have modified the difficulty of the reaction time task between participants or increased the distance between successive targets. Contact sensors, however, are commercially available, accurate, and increasingly common in PD research (73). Researchers may therefore consider using this technology for objective motor assessment.

Multiple studies assessed manual dexterity using the 9-hole peg test (29, 30). This paradigm is frequently used in the PD literature, allowing for comparisons to other intervention types. Future researchers may also consider using the Purdue pegboard test, which likewise is well validated for use in PD intervention studies (74).

In general, future researchers may consider using wearable and digital technology that is objective, accessible, deployable by research assistants with a broad range of experience and training, and comparable to existing datasets.

### Limitations

This systematic review is limited by the relative paucity of publications examining climbing as a therapeutic intervention for PD. To minimize the effects of this anticipated limitation, broad search terms were selected, reference chaining was used, and broad inclusion criteria were set with no additional exclusion criteria. Searches were conducted in large, internationally used databases, but may have missed work only searchable in its original language. Only articles published in peer-reviewed journals were included in this review, excluding unpublished work such as conference posters and proceedings. This may have contributed to the limitingly small number of eligible references Additionally, this review was not pre-registered. Registration of reviews can assist researchers in minimizing bias (75). As this review was not registered, there was no published protocol available for this purpose. However, once the search strategy was formalized, no changes were made throughout the duration of the review process.

Both authors of this review are recreational climbers and one of the article authors operates a nonprofit that facilitates a climbing program for people with PD. This involvement in the climbing community may have led to implicit pro-climbing bias. This limitation was addressed through the use of a rating scale for quality assessment applicable to multiple types of exercise, rather than unique to climbing, as well as the use of independent evaluation by two authors during the quality assessment process. Future researchers may mitigate this potential bias by including climbing-naïve authors and by examining the feasibility of clinicians without climbing expertise implementing climbing-based interventions.

The included literature explored the efficacy of climbing as an intervention for only a small demographic range of all patients with Parkinson's disease. In the composite sample, only about one third of participants were female. Furthermore, the study by Ries et al. was the only publication that presented demographic data on race or education, and 85.7% of participants identified as white, and 89.3% had a bachelor's degree or higher (29). These demographic factors may be associated with sociocultural characteristics favoring positive exercise habits and pre-intervention perception of climbing as a safe and desirable activity for them and their peers. For example, college education is associated with increased exercise adherence among older adults (76). Additionally, climbing is often perceived as a male- and white-centric form of exercise (77, 78). Future investigators should evaluate the feasibility of enrolling participants from diverse backgrounds and acceptability of climbing for diverse populations of people with PD.

Eligible articles were assessed using the Methodological Quality of Exercise Training Studies Scale. This scale was introduced by Brughelli et al. in 2008 (26) and has since been used in multiple review articles (27, 79). This tool was selected for its good face validity and flexibility to multiple methodologies. A limitation of the Methodological Quality of Exercise Training Studies Scale, however, is that it has not been validated against other tools or analyzed for inter-rater reliability. Furthermore, it is a quality evaluation tool, not strictly a risk of bias assessment. The items in the Methodological Quality of Exercise Training Studies Scale do, however, use the same 0–2 rating system as the Methodological Index for Non-randomized Studies (MINORS)—a validated risk of bias assessment. The MINORS is applicable for use in single-arm non-randomized studies, which is the design of the two non-randomized studies included in this review (80). Furthermore, the items of the Methodological Quality of Exercise Training Studies Scale generally map onto the domains of the MINORS. The item “participant inclusion criteria were clearly stated” item maps onto the domain “inclusion of consecutive patients.” The items “a control group was included” and “participants were assigned to groups randomly and groups had equal baselines,” map onto the MINORS domain “adequate control group” and “baseline equivalence of groups.” “The intervention was thoroughly described” aligns with the MINORS domain “prospective collection of data.” The items “dependent variables were defined” and “assessment protocols were defined and practical” map onto the domain “endpoints appropriate to the aim of the study.” “Statistical tests were applied appropriately” and “results were presented in detail and include values from statistical tests” aligns with the MINORS domain “adequate statistical analyses.” The item “training duration was practical” maps onto “follow up period appropriate to the aim of the study.” The exercise scale item “conclusions were insightful” does not map neatly onto a MINORS domain, and the MINORS domains “clearly stated aim,” “unbiased assessment of study endpoint,” “prospective calculation of study size,” “lost to follow-up less than 5%,” and “contemporary groups” were not addressed by the quality assessment applied in this review.

Although there are conceptual similarities between the items, the lack of direct application of a validated risk of bias assessment is a substantial limitation of this review. Future systematic review authors may improve risk of bias evaluation by using a validated risk of bias tool appropriate to the methods of interest, such as the MINORS or ROBINS-I (28, 80). Future reviewers may also consider using a validated exercise-specific tool such as the Tool for the assEssment of Study qualiTy and reporting in EXercise (TESTEX) (81).

### Future directions

Climbing-based interventions included in this review demonstrated validity and favorable initial results that warrant further investigation. This investigation should include both qualitative examinations of participant experiences, as well as quantitative, standardized, and digital measures of motor and non-motor outcomes. Future randomized controlled trials may compare climbing to other established exercise programs, as well as examine the effects of climbing duration and intensity on outcomes of interest to optimize exercise recommendations.

Researchers should also use rigorous methods to evaluate participant experiences and perceptions of climbing. Future qualitative research should limit participant and researcher bias, perhaps by including interviews with open-ended questions. Understanding participant experiences may aid in refining interventions to promote positive experiences and therefore participant adherence.

An improved understanding of the physiological response to climbing among people with PD may aid in developing an optimal exercise prescription. Exercise physiologists have examined the cardiovascular and metabolic effects of climbing; however, these studies were conducted with neurologically healthy athletes and recreational climbers (20, 55, 82). Examining physiological responses in people with PD may help integrate climbing into comprehensive exercise prescriptions. Future investigations may include using wearable heart rate and metabolic monitors, which have been validated for use in people with PD and for research into the physiology of climbing (65, 82).

A major gap in the current literature is the absence of comparisons of non-motor symptoms before and after climbing interventions. Future researchers may use validated assessment paradigms and surveys to report the effects of climbing interventions on these symptoms and allow for comparisons to other exercise programs and intervention types (65). Additional research in the motor domain may also use established outcomes to allow for comparisons between exercise types and intervention protocols.

## Conclusion

Climbing is a safe and acceptable form of exercise that may be beneficial for people with PD. Climbing has good face validity compared to current exercise recommendations for people with PD because it involves cardiovascular, strength, flexibility, and neuromotor elements. The literature examining the effects of climbing on PD symptoms is small but emerging. Climbing-based interventions were associated with improvements in motor performance—including increased gait speed, increased lower extremity strength, improved balance, increased manual dexterity, and more upright posture. Participants in a climbing intervention demonstrated a greater decrease in motor symptoms in each of the three MDS-UPDRS-III subdomains—bradykinesia, tremor, and rigidity—than an unsupervised exercise control group. With the exception of the dexterity and some locomotor tasks, assessments were only conducted in one publication, and the results have not been verified. This limits the strength of the current evidence base. Further research with strong methodologies is warranted to validate and expand upon the current results. This research should aim to optimize guidelines for climbing, quantitatively examine effects in the non-motor domain, and utilize validated, technology-based, and objective measurements that allow within-study and meta-analytic comparison to other exercise types and treatment approaches. Meanwhile, people with PD may safely participate in, and likely experience benefits from climbing, especially top-rope SC.

## Data Availability

The original contributions presented in the study are included in the article, further inquiries can be directed to the corresponding author.
